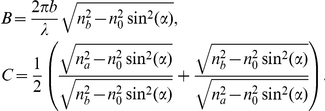# Negative Refraction Angular Characterization in One-Dimensional Photonic Crystals

**DOI:** 10.1371/journal.pone.0017188

**Published:** 2011-04-06

**Authors:** Jesus Eduardo Lugo, Rafael Doti, Jocelyn Faubert

**Affiliations:** Visual Psychophysics and Perception Laboratory, School of Optometry, University of Montreal, Montreal, Quebec, Canada; Joint Research Centre - European Commission, Germany

## Abstract

**Background:**

Photonic crystals are artificial structures that have periodic dielectric components with different refractive indices. Under certain conditions, they abnormally refract the light, a phenomenon called negative refraction. Here we experimentally characterize negative refraction in a one dimensional photonic crystal structure; near the low frequency edge of the fourth photonic bandgap. We compare the experimental results with current theory and a theory based on the group velocity developed here. We also analytically derived the negative refraction correctness condition that gives the angular region where negative refraction occurs.

**Methodology/Principal Findings:**

By using standard photonic techniques we experimentally determined the relationship between incidence and negative refraction angles and found the negative refraction range by applying the correctness condition. In order to compare both theories with experimental results an output refraction correction was utilized. The correction uses Snell's law and an effective refractive index based on two effective dielectric constants. We found good agreement between experiment and both theories in the negative refraction zone.

**Conclusions/Significance:**

Since both theories and the experimental observations agreed well in the negative refraction region, we can use both negative refraction theories plus the output correction to predict negative refraction angles. This can be very useful from a practical point of view for space filtering applications such as a photonic demultiplexer or for sensing applications.

## Introduction

Photonic crystals can be considered as multidimensional periodic gratings, in which the features of refraction at flat surfaces are dominated by Bragg diffraction effects. The refraction angle from positive to negative can be tailored based on photonic band theory [1]. Numerous studies on diffraction gratings and periodic planar waveguides, essentially the one-dimensional counterparts for the photonic structures, led to the observation of a vast variety of anomalous refraction effects, including “birefringence” [2]–[6]. These systems have undergone extensive and systematic study based on the wave vector diagram formalism. This formalism has proven to be an excellent tool in explaining the unusual refractive properties for the one-dimensional diffraction grating system. In the late 1990s, diffraction characteristics that appeared to be negative refraction were explained in terms of the dispersion surfaces of photonic bands and prism, lens, and collimation effects based on refraction were predicted [7]–[9]. Specifically, it has been demonstrated that light propagation in strongly modulated 2D/3D photonic crystals becomes refraction-like in the vicinity of the photonic bandgap, even in the presence of strong multiple diffraction [4]. In these conditions, it is possible to define an effective phase refractive index to explain the propagation inside the photonic crystal using the conventional Snell's law. Since such effective index is determined by the photonic band structure, it can be negative and less than unity, which leads to negative refraction [9].

This behavior can be understood by using the effective-mass model in electron-band theory. In the photonic case a Bloch photon, near the bandgaps, can be considered as free, and be regarded as a refracted photon inside of a medium with an effective refractive index. These particular index states only appear close the photonic bandgap in a similar way as the effective mass states in a semiconductor. The same conclusion has been reached by others groups [10]. For instance, the effective dielectric constant of a 2D photonic crystal in all optical bands, for both TE and TM polarizations, was calculated. It has been found that near the gamma point (center of the Brillouin zone), the dispersion relationship for the TM mode is independent of the propagation direction, while the TE mode in general depends on the electromagnetic waves propagation direction. Therefore, for a 2D photonic crystal, there always exists an effective dielectric index for the TM mode near the gamma point. However, it cannot be defined as an effective refractive index for TE mode unless the photonic crystal is highly symmetric. By using similar arguments presented in [9], Kavokin theoretically explored negative refraction in one-dimensional photonic crystals (1D PCs) [11]. By using the dispersion of the photonic bands, he inferred negative refraction zones from frequency regions where the effective mass is negative. Recently, we have simulated a lossless 1D PC structure and showed that negative refraction could be present near the low frequency edge of at least the second, fourth and sixth bandgaps [12]. The same conclusion was reached by other groups [13]–[15]. Furthermore, we experimentally demonstrated negative refraction in strongly modulated porous silicon 1D-PC in the visible and near infrared regions. However, in [12] negative refraction was explored with only one angle of incidence. Therefore, a complete angular characterization is still missing.

Moreover, in regards to the theory of negative refraction in 1D PCs, the existence of antiparallel energy and phase velocity has been thoroughly analyzed in [16]. The existence of negative refraction in 2D PCs is substantially different from the one-dimensional case because 2D PCs with a negative slope band demonstrates negative refraction beam propagation. This is not true for 1D PCs because the correctness propagation condition needs to be fulfilled. The “correctness” of propagation in 1D PCs implies that the correct physical conditions, required to observe negative refraction, are met. The analysis presented in [16], for 2DPCs, only tackles negative refraction for on-plane propagation where the crystal is periodic. The exact analogy for negative refraction propagation between 2D PCs and 1D PCs is the normal incidence case, where the 1D PCs are periodic in that particular direction. Nonetheless the aforementioned correctness condition should also be applied in the 2D PCs case when you have off-plane propagation, a point that we will discuss later.

In this paper, we experimentally completed the angular characterization of negative refraction in a 1D PC structure, near the low frequency edge of the fourth photonic bandgap. We compared the experimental results with current negative refraction theory in 1D PCs [11] and with a theory developed here, based on the group velocity. We confronted both negative refraction theories and found good agreement between them with differences up to 4 degrees, within the explored incidence angle interval. We analytically derived the correctness condition and showed that for the experimental conditions we used, the correctness condition is fulfilled up to an incidence angle of 15 degrees. We also theoretically verified the correctness condition near the second bandgap edge (1350 nm) and found that it is fulfilled up to an incidence angle of 20 degrees. In order to compare the experiments with theory we developed an approximation that accounts for the positive refraction that the negative refraction beam suffers at the structure output. The correction uses Snell's law and an effective refractive index, based on two effective dielectric constants [17]. We found good agreement between experimental observations and the theory developed here for the whole incidence angle interval explored. The agreement between current theory and experimental results was good for incidence angles smaller than 15 degrees because the effective mass approximation begins to fail for incidence angles larger than 15 degrees and so does its consequent correction approximation. Since both theories and experimental results agreed well in the negative refraction region, given by the correctness condition, we can use both negative refraction theories with the addition of the output correction given herein to predict negative refraction angles.

## Results and Discussion

### Sample preparation and negative refraction angle for the output measurement

**Figure 1:**
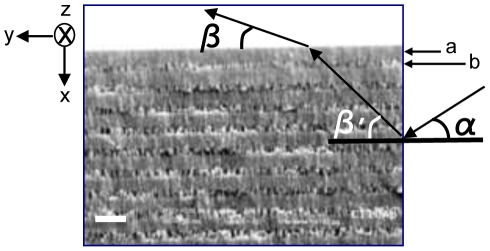
1D PC structure. SEM picture showing the layers a and b, angle of incidence α and negative refraction angle β^′^ inside the structure and corrected negative refraction angle β at the output, which can be measured experimentally. The light impinges at the right interface (The white line on the left represents 1 micron).

Porous silicon (Psi) multilayers (Fig. 1) were prepared by electrochemical anodization of crystalline silicon (c-Si) [18]. Porous silicon was fabricated by wet electrochemical etching of highly boron-doped c-Si substrates with orientation (100) and electrical resistivity of 0.001–0.005 Ohm-cm (room temperature = 25°C, humidity = 30%). On one side of the c-Si wafer, an aluminum film was deposited and then heated at 550°C during 15 minutes in nitrogen atmosphere to produce a good electrical contact. In order to have flat interfaces, an aqueous electrolyte composed of HF/ethanol/glycerol was used to anodize the silicon substrate. It is well known that the Psi refractive index increases by decreasing the electrical current applied during the electrochemical etching. However, reducing the porosity too much might stop the electrolyte flow through the porous and limit the subsequent high porosity layer that makes the contrast. One way to allow the electrolyte to flow is by increasing the ethanol fraction in the solution. For this reason, an electrolyte composition of 3∶7∶1 was used. In addition, the HF concentration was maintained constant during the etching process using a peristaltic pump to circulate the electrolyte within the Teflon™ cell. Anodization begins when a constant current is applied between the c-Si wafer and the electrolyte by means of an electronic circuit controlling the anodization process. To produce the multilayers, current density applied during the electrochemical dissolution was alternated from 3 mA/cm^2^ (layer a) to 40 mA/cm^2^ (layer b) and eighty periods (160 layers) were made. Psi samples were partially oxidized at 350°C for 10 minutes. The best refractive index values we found that fit the experimental photonic bandgap structure studied here are 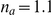 and 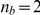 [12]. We have experimentally measured the refractive indices of single Psi layers made with the same electrochemical conditions as the multilayers [18] and we found that 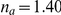
±0.07 and 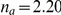
±0.11. The refractive indices were measured by using interference fringes from reflectance measurements [18]–[19]. Nevertheless, it is known that the refractive index and etching rate for a single layer are modified in the presence of a multilayer structure up to approximately 14%, a phenomenon that has been systematically observed [19]. This result might have the consequence of compromising the mechanical stability of the structure. Indeed, in certain regions seen in Fig. 1 layers appear to be collapsed. Nevertheless, negative refraction was observed in all our experiments where several regions were scanned. Scanning electron microscopy (SEM) was used to measure the films thicknesses which were 326±11 nm (*a*) and 435±11 nm (*b*).

**Figure 2:**
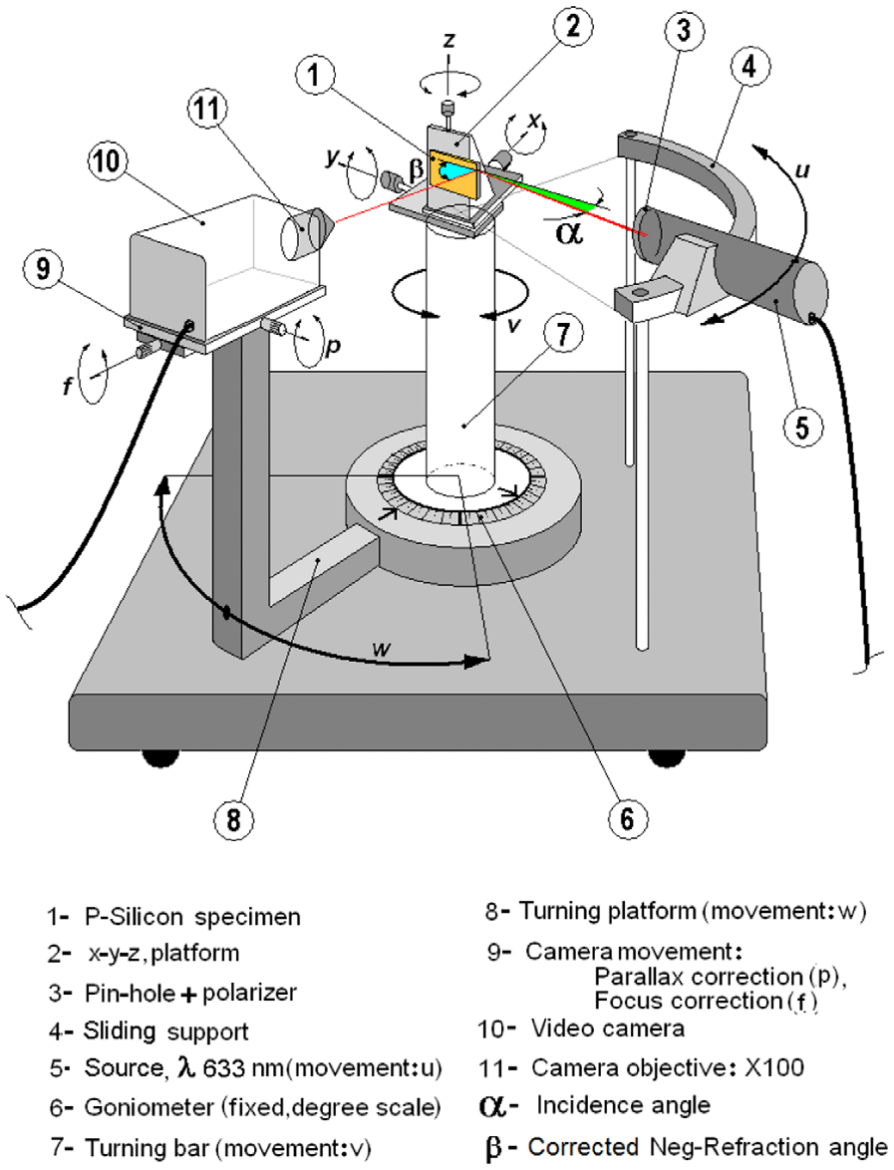
Experimental set up. The eleven components of the experimental setup for negative refraction observation.

Once the samples were ready, we investigated the relationship between the negative refraction angle and the incidence angle at 633 nm (TE polarization) for the 1D PC structure. Figure 2 shows the experimental setup we used. The apparatus consists of a plate on which we find a *curved support with a sliding base (4)*, a *turning bar (7)* and a *turning platform (8)*. There is a *light source of 633 nm (5)* that can slide on the *curved support (4)* that points towards the turning center. On top of the *turning bar (7) we* find a *xyz platform (2)* that holds the *1D PC (1)* under test. The *turning platform (8)*, which holds the video camera (10), has *two movement axes (9)*. These materials were placed on a standard optical table. We illuminated the sample edge with a light source at the desired incidence angle 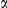 and, by exploring the sample side with the video camera; we found the output refracted beam (corrected negative refraction angle 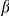). Once the beam was detected, its direction was confirmed by means of the beam spot luminance on the image monitor (not shown) that was measured with a luminance meter. As the refracted beam gets weaker for higher incidence angles, we explored angles up to 25 degrees in order to have enough discrimination of the spot luminance in reference to the monitor image background luminance. More details are given in the methods section.

### Negative refraction theory

In order to compare the experimental corrected negative refraction angles at the output (angle 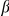) with theory, first we need to discuss negative refraction theory that allows us to calculate negative refraction angles 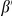 as a function of incidence angles 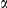 (see Fig. 1). Negative refraction theory for 1D PCs has been presented in reference [11], where the condition for negative refraction uses the notion that, if in a given direction the effective mass is negative, the corresponding components of group and phase velocities of light have different signs. This seems to be true for 1D PCs because they are strongly anisotropic, so that the effective masses have different signs in on-plane and normal-to-plane directions [11]. However, in order to fully warrant the occurrence of negative refraction the correctness condition needs to be fulfilled [16].

**Figure 3:**
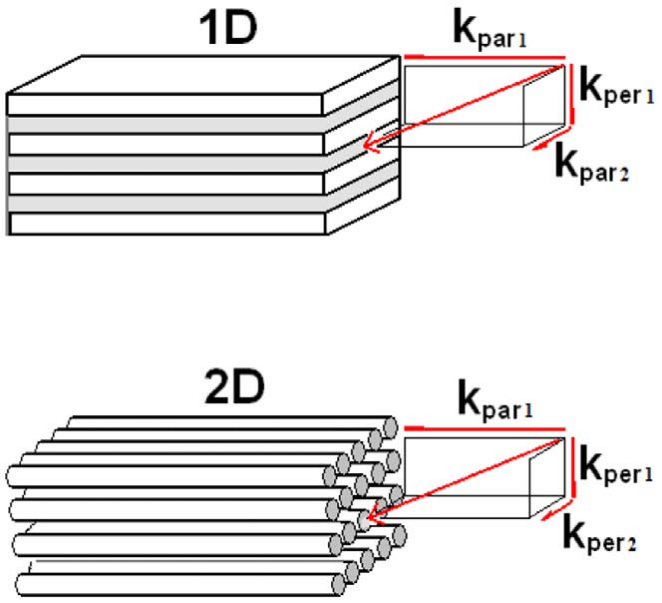
Correctness condition. Correctness condition generalization from 1D PCs to 2D PCs.

It is well known that there are significant differences between the properties of 1D PCs and 2D PCs. In 2D PCs, when the plane of incidence is chosen to be the periodic plane, the entire wave vector is confined in the first Brillouin zone. In the theory of wave propagation through a crystal lattice, the Brillouin zone is a fundamental region of wavevectors; every vector outside this region is tantamount to some other vector inside it. In contrast, in the 1D PC, only the component of the wave vector along the direction of periodicity is restricted within the first BZ. This has a very important implication. In 2D PCs, a band with negative slope corresponds to a negative refraction beam. However, this is not true for 1DPCs. We have chosen *x* to represent the direction of the periodicity (Fig. 1). The slope of a certain band will then be given by 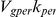, where 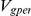 and 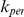 are the group velocity and wavevector components in the normal-to-plane direction respectively. Since 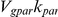 is always positive, where 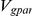 and 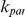 are the group velocity and wavevector components in the on-plane direction respectively, and for a band with positive slope 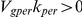, then 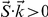, where 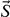 and 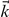 are the Poynting vector and the wavevector respectively. For a band with negative slope 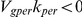, then the correctness condition for negative refraction to occurs 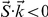 gives
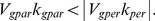
(1)Where we have used the fact that the Poynting vector is proportional to the group velocity. The group velocity components 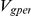 and 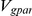 can be obtained from the photonic bands' dispersion relationship as outlined in [13] as:
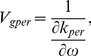
(2)

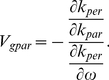
(3)We have verified condition 1 (see the methods section), for our proposed 1D PC structure, close to the second (1350 nm) and fourth (633 nm) low frequency band edges (TE Polarization) and used refractive index values and layer thickness described in the experimental section and with 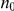 equals one. Condition 1 is fulfilled for incidence angles up to 20 degrees and 15 degrees respectively. For 633 nm light, traveling in the structure, one should expect that for angles of incidence larger than 15 degrees there will be more than one beam travelling inside the structure. For the normal-to-plane direction the second and fourth allowed band ends at 1345 nm and 630 nm respectively and they are characterized by a negative parabolicity close to the band edge. On the other hand, for the on-plane direction it is also parabolic close to band edge but it is characterized by a positive effective mass. In the case where the relevant bands have different band slope signs, one can observe the simultaneous propagation of beams [16]. Condition 1 can be generalized as
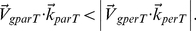
(4)Where 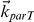 and 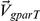 are the total wavector and group velocity in the parallel direction. 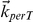 and 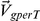 are the total wavector and group velocity in the perpendicular direction. In the 1D PC case the vectors, according with figure 3, are given by 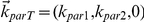, 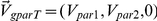, 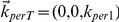, 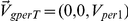 and in the 2D PC case by 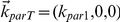, 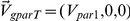, 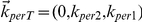 and 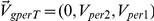.

It is clear that if we are in a band with a negative slope where 
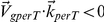
 is always true and since in the parallel direction 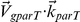 is always positive. Therefore inequality 4 gives the negative refraction correctness condition either for 1D PCs or 2D PCs. The parallel direction represents the direction where there are no periodic dielectric regions to coherently scatter the light. For instance, in a 1D PC is the on-plane direction (known as off-axis as well) and for a 2D PC is the off-plane direction.

The expression for the negative refraction angle 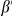 for the geometry showed in figure 1 is obtained in [11] by using the continuity of the electric and magnetic fields at the boundary and the effective mass approximation as:
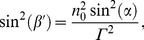
(5)where 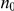 is the air refractive index, 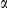 is the incidence angle, c is the speed of light, 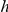 is Planck's constant, 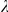 is the working light wavelength, 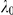 is the wavelength associated with the top of the fourth subband, for instance and 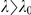. The effective mass approximation works fine only if the condition 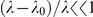 is fulfilled. The parameter 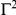 is expressed as
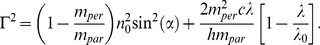
(6)The effective masses of light in the normal-to-plane direction 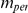 and on-plane direction 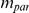 are calculated by using the expressions given in the methods section.

**Figure 4:**
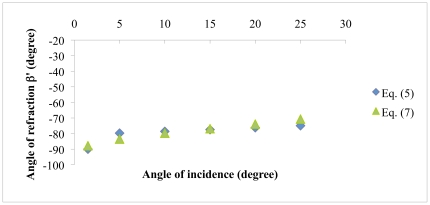
Negative refraction theories comparison. Angle of refraction β′ vs. angle of incidence for the 1D PC proposed structure. The theory presented in Kavokin [11] is compared against group velocity theory. The light wavelength is 633 nm (TE polarization) and we used refractive index values and layer thickness described in the experimental section.

We can also calculate negative refractive angles by using the group velocity, which represents the direction of propagation inside the medium as follows:
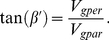
(7)Equation (7) is given in the methods section. Figure 4 shows the comparison between Eqs. (5) and (7). We used a working wavelength of 633 nm (TE Polarization) which is close to the fourth low frequency band edge (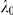-630 nm), and we used refractive index values and layer thickness described in the experimental section with 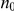 equals one. Clearly both curves are similar with angle value differences up to 4 degrees, within the explored incidence angle interval. This result supports the use of the theoretical approach represented by Eq. (5) to predict negative refraction angles.

### Comparison between experimental results and theory

**Figure 5:**
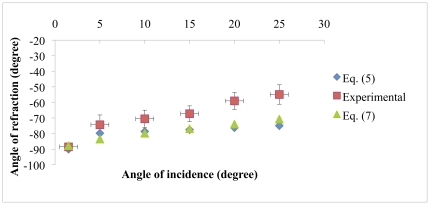
Comparison between negative refraction theories and experiments. Negative refraction experimental values compared against uncorrected theoretical values (Eqs. (5) and (7)).

Figure 5, shows the experimental and negative refraction results for the theories (eqs. (5) and (7)) for the behavior between angle of incidence versus angle of refraction. Since the experimental values represent corrected negative refraction angles at the output (angle β) we cannot compare them directly with the theories because they represent negative refraction angles β′ inside the structure.

**Figure 6:**
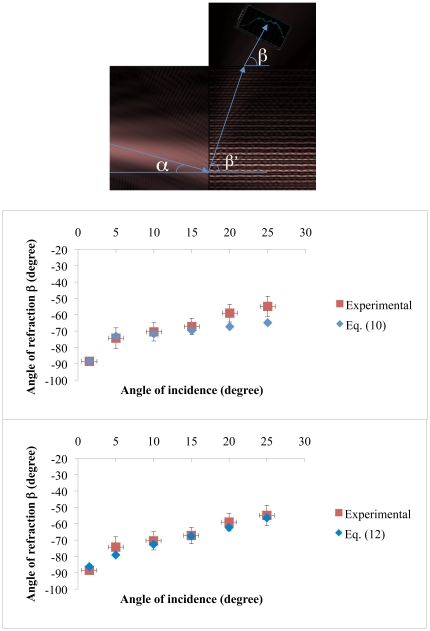
1D PC results at a working wavelength of 633 nm (TE polarization). (Top) finite element negative refraction simulation showing beam propagation inside the structure and input and output interfaces. The angle of incidence α is 15 degrees, angles β′ and β are −72 and −63 degrees respectively. (Middle) Comparison between negative refraction experimental values and corrected theoretical values (Eq. (10)). (Bottom) Comparison between negative refraction experimental values and corrected theoretical values (Eq. (12)). We used refractive index values and layer thickness described in the experimental section. Error bars represent systematic errors plus random errors (two standard deviations).

Reference [16] investigated light propagation in a 2D PC that consisted of dielectric rods in air with a hexagonal arrangement for the *H*-polarization case. They first performed a finite-difference time-domain (FDTD) simulation of light propagation along the Γ-K interface with an incidence angle of 8 degrees. The Γ-K interface goes from the center of the Brillouin zone to a vertex that joins two edges. Second, they supposed that their periodic structure could be described with an effective medium having an effective dielectric constant consistent with Maxwell-Garnett theory [16] and, therefore, an effective refractive index. In such a case, the field inside the PC is a plane wave. Third, by using the plane wave expansion method (PWE) they determined that for low dielectric contrasts between rods and air there is mainly one predominant component contributing to the Floquet-Bloch wave (FB). If the dielectric contrast between rods and air is bigger than 2, mixing between the different components in the FB sum starts to occur. This was corroborated by their FDTD simulations. Fourth, for both treatments, the effective homogeneous medium and the periodic medium with the PWE method gave almost the same angle for the propagating beam. This value is in excellent agreement with the FDTD simulation result. Given this, one might think that it is possible to describe a photonic crystal medium, for low dielectric contrast, as a homogeneous medium with an effective index. However, if you take the same angle of incidence, but choose a different interface such as Γ-M. The Γ-M interface goes from the center of the Brillouin zone to the middle of an edge. The propagation results are completely different to the precedent case and cannot be described by a homogeneuos medium approximation. From this we can infer that the wave is able to see the periodicity of the medium even when the index contrast is low. Nonetheless, the fact the effective medium approach fails to generally describe beam propagation in some cases, this does not preclude the use such approximation to describe beam propagation in a particular direction if there is only one predominant FB wave travelling in that direction. We have done a finite element simulation of our structure (figure 6-top) where we can observe a single negative refraction beam (beam with angle β′) that impinges towards the normal-to-plane interface where it is positively refracted as a single beam. This implies that we can use an effective medium approximation in such direction but we have to bare in mind that the effective refractive index does not represent the refractive index of the structure as if it were a homogeneous medium in all directions.

Since the negative refraction beam does not only propagate in the normal-to-plane direction we have to construct an effective medium approximation that takes into account the on-plane propagation direction as well. We can use the normal-to-plane 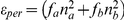 and on-plane 
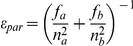
 effective dielectric constants, known to work fine in a multilayer system [17]. Therefore we can construct an effective medium approximation with an effective index of refraction given by
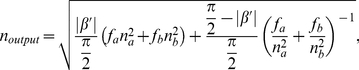
(8)where 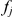 represents the fraction volume of each layer (43% for a-layers and 57% for b-layers), and 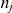 is the refractive index of each layer. The two angular prefactors multiplying each dielectric bound are necessary to account for the contribution of each component (normal-to-plane and on-plane). Then we can use Snell's law and the effective refractive index as:
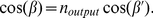
(9)Combining equations (5), (7), (8) and (9) the corrected negative refraction angles at the output can be calculated as:
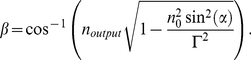
(10)Equation (10) is valid for 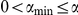, where 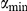 is given by
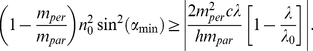
(11)Inequality (11) assures us that the angle 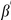 is real.

Now, by combining equations (7), (8), and (9) the corrected negative refraction angles at the output can be calculated as well as:
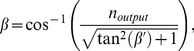
(12)and the analytical expression for 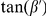 is given in the methods section. Clearly, Eq. (5) values fit well with experimental values up to 15 degrees (fig. 6-middle). The angular difference 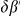 between values predicted by eqs. (5) and (7) is the reason why the corrected refraction angles at the output, obtained by using eq. (10), differs from the experimental ones for angles of incidence larger than 15 degrees. This is understandable because as the incidence angle increases the effective mass approximation begins to fail. Indeed by increasing the incidence angle, the band edge is pushed towards small wavelengths making the separation 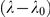 increase. The results for the corrected negative refraction angles obtained with eq. (12) are shown in figure 6, (bottom). Notice that equation (12) predicts values that lie within the experimental accuracy obtained for all the angles of incidence. Notwithstanding, equation (10) is a good approximation to calculate corrected negative refraction angles at the output and it works well in the negative refraction region given by the correctness condition. All the experiments and calculations were done for TE polarization and a similar approach can be used for TM polarization where we expect to find analogous results as it was shown in reference [12].

### Conclusion

In conclusion, we have experimentally completed the angular characterization of negative refraction in a 1D PC structure, near the low frequency edge of the fourth photonic bandgap and compared it with current theory and theory based on group velocity developed here. We have validated the current negative refraction theory approach with our theory. We found good agreement between both theories with differences within 4 degrees in the explored incidence angle interval. In order to know the negative refraction zone, we have analytically derived the correctness condition and showed that for the experimental conditions we used, the correctness condition is fulfilled up to an incidence angle of 15 degrees. We also theoretically verified the correctness condition near the second bandgap edge (1350 nm) and found that it is fulfilled up to an incidence angle of 20 degrees. Finally, we corroborated the angular experimental values with negative refracted angular values obtained with both negative refraction theories by applying an output correction that uses Snell's law and an effective refractive index, based on the two effective dielectric constants. We found good agreement between experimental results and our theory in the entire incidence angle interval explored. The agreement between current theory and experimental results was good up to an incidence angle of 15 degrees because the effective mass approximation begins to fail for incidence angles larger than 15 degrees and the same is true for its consequent correction approximation. Since both theories and the experimental observations agreed well in the negative refraction region given by the correctness condition, we can use the combination of theory and output correction to predict negative refraction angles. This is very useful from a practical point of view. For instance, it could be useful for space filtering applications [20] such as a photonic demultiplexer or for sensing applications. A demultiplexer could be based on the fact that it is possible to have different wavelengths light impinging on the same incidence angle, since β′ depends on the wavelength, light with different wavelengths is dispersed in different directions at the output. Equations (10) and (12) will consequently be useful to estimate the output angles. A (Bio)chemical sensor could instead exploit the fact that the multilayers are porous and we can change their refractive indices by infiltrating different chemical or biological compounds that again would shift the angles β′ and β. Compound concentration should be proportional to this angular shift.

## Materials and Methods

### Determination of the refracted angle 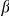: step sequence (see Fig. 2)

The first step consisted on choosing a convenient position of the light source on the sliding support. That was chosen in function of the free space needed for hand intervention. Once this position was determined, it was kept invariant along all the measurements. The light beam was kept as angular reference for zero degrees. So, through the *turning movement v* and the linear *displacement x*, the second step consisted in obtaining a regular tangent light beam observed all along the lateral face of the specimen (parallel to *y* direction). The third step was to assure that the specimen illuminated edge was placed just over the turning center of the apparatus. This task was performed by acting the *movement y*. The fourth step was moving the *turning bar (7)* around to place the 1D-PC in the desired incident angle 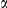 in reference to the light beam. To achieve this, we acted the *movement v* and verified the angular position on the *goniometer (6)* scale. At this point, it was necessary to place the 1D-PC specimen in a way that assured us that the incident beam was totally contained in the illuminated edge, and without reaching the specimen normal face (*x* direction). This was done by means of the *movement x* (sixth step). Then we explored the specimen side looking for a negative-refracted beam by means of the video camera, the turning *platform* and controlling the parallax error (by keeping the refracted light spot centered on the TV monitor and in focus). This seventh step involved the movements: *w*, *p* and *f*. After we found the light spot, we explored a narrow angle 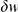 maintaining the light spot centered in the monitor, as we explained before. Using a luminance-meter (measure of the luminous intensity of light travelling in a given direction) we controlled the light level emitted by the monitor in the portion of the image containing the refracted light spot. With this procedure we found the angle 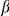 for which the luminance-meter gave the highest reading (*Lmax*), and then we checked the refracted intensity for points five degrees away from this last one, verifying that their intensity was less than *50% of Lmax*. This tedious procedure was repeated for incident angles ranging from 1.5 degrees up to 25 degrees. Each angle was measured four times, but we reported the average value as the negative refraction value and two standard deviations errors as random errors.

The transference from the refracted light intensity (that we expect to follow a Gaussian-like distribution according to our simulations [12]) to the monitor emitted light (measured with the luminance meter), cannot be considered proportional because of the energy conversions involved (all with their own non linearities and convergence limits). The narrow intensity per unit of area distribution of the refracted light and the acceptance angle of the camera suggested that the most important systematic error was due to two factors. First, the angular determination error: angular measurement through mechanic goniometers could reach without problems 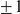 degree error; but the *Lmax* reading gave us a non discernible reading along 3 degrees around the *Lmax* 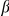 angle. This effect is known as spatial filtering. Second, we explored the negative refracted spot light along a circumference centered in the same spot as if it where the center for the *w* movement. Unfortunately the real center (for the turning platform) and the refracted spot was several microns away (at least the distance from the spot to the specimen edge). Therefore, a further correction due to the parallax and eccentricity compensation is needed to solve this problem. Once more, as the refracted beam presented a narrow distribution and the *Lmax* gave us a 3 degree error, this was covered largely other systematic errors involved. We used the same light source and polarizer reported in [12] and the negative refractive transmitted light was captured by a CCD camera (KP-D50, Hitachi)) coupled with a singlet lens (focal length of 8 mm, NT-45114, Edmund Optics) placed at 8 mm from the sample. The signal from the camera was sent to a color analogical monitor and a luminance-meter (CS-100, Minolta) was placed at 50 cm from the monitor.

### The correctness condition can be expressed as



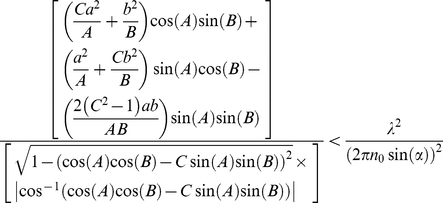
where
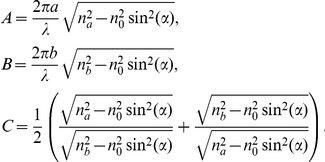



### Effective mass approximation expressions

The effective mass expressions that only work close to the band-edge can be obtained from reference [11] and are given by:
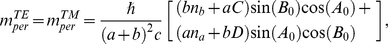


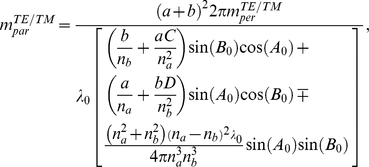
where  
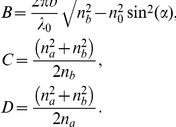


The signs “−” and “+” in the expression for 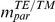 correspond to TE and TM polarized light respectively.

### Equation (7) expression



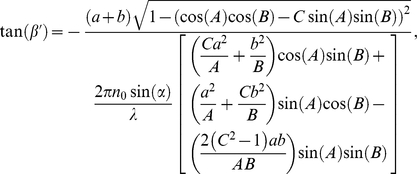
where